# Review on the Role of Epigenetic Modifications in Doxorubicin-Induced Cardiotoxicity

**DOI:** 10.3389/fcvm.2020.00056

**Published:** 2020-05-07

**Authors:** Himani Kumari, Wan-Hong Huang, Michael W. Y. Chan

**Affiliations:** ^1^Department of Biomedical Sciences, National Chung Cheng University, Chiayi, Taiwan; ^2^Epigenomics and Human Disease Research Center, National Chung Cheng University, Chiayi, Taiwan; ^3^Center for Innovative Research on Aging Society (CIRAS), National Chung Cheng University, Chiayi, Taiwan

**Keywords:** cardiotoxicity, chemotherapy, epigenetics, cancer, doxorubicin

## Abstract

Use of anthracyclines such as doxorubicin (DOX), for the treatment of cancer, is known to induce cardiotoxicity, begetting numerous evaluations of this adverse effect. This review emphasizes the mechanism of how consideration of DOX-induced cardiotoxicity is important for the development of cardioprotective agents. As DOX is involved in mitochondrial dysfunction, enzymes involved in epigenetic modifications that use mitochondrial metabolite as substrate are most likely to be affected. Therefore, this review article focuses on the fact that epigenetic modifications, namely, DNA methylation, histone modifications, and noncoding RNA expression, contribute to DOX-associated cardiotoxicity. Early interventions needed for patients undergoing chemotherapy, to treat or prevent heart failure, would, overall, improve the survival, and quality of life of cancer patients. These epigenetic modifications can either be used as molecular markers for cancer prognosis or represent molecular targets to attenuate DOX-induced cardiotoxicity in cancer patients.

## Introduction

Cardiotoxicity, in simpler terms, is defined as “toxicity which damages the heart,” often during or after chemotherapeutic treatment (1). Treatment options for cancer have been improving significantly in recent years, and the rates of survival in several human cancers have increased significantly with reduced recurrences (2). However, the applicability of these drugs is limited by the risk of cardiotoxicity (1). Doxorubicin (DOX)–induced cardiomyopathy can occur within a few days of its administration or delayed until decades after chemotherapy, thus affecting morbidity, mortality, and quality of life of cancer patients (3–6). However, the mechanism of DOX-induced cardiotoxicity is not fully understood.

Epigenetic modifications, including DNA methylation, histone modifications, and noncoding RNA (ncRNA) expression, play an important role in regulating gene expression and are considered as a hallmark of several human diseases, such as cardiovascular disease [review in Kimball and Vondriska (7)]. In this review, we discuss the mechanism of how aberrant epigenetic modifications contribute to DOX-induced cardiotoxicity and possible alternative therapeutic options that could forestall or prevent chemotherapy-induced cardiotoxicity (8).

## Chemotherapeutic-Associated Cardiotoxicity

In the 1960s, DOX (Adriamycin®), first isolated from *Streptomyces* actinobacteria, was found as one of the first anthracyclines (9), to be used for several cancer treatments, including breast carcinomas, sarcomas, leukemias, non-Hodgkin and Hodgkin lymphoma, and many other cancers (10, 11). At the molecular level, DOX acts to stabilize topoisomerase DNA isomers and therefore blocks DNA replication and transcription (12, 13). It has been reported in several studies over the last 15 years that despite the successful development of small molecules and targeted therapies, anthracycline-based chemotherapy still plays not only prominent anticancer but also overall detrimental roles in many types of cancer treatment (14). Concerning the latter, DOX causes a cumulative, irreversible, and dose-dependent cardiomyopathy that ultimately leads to congestive heart failure (15). Previous studies have demonstrated that cardiotoxicity is a repercussion of dose-dependent administration of DOX, with those exceeding 500 mg/m^2^ greatly increasing the risk of congestive heart failure tremendously (16). Understanding the mechanism involved in DOX is important in developing novel preventive measures, and treatment strategies, against DOX-induced cardiotoxicity.

Cardiotoxicity is one of the major adverse effects of chemotherapy, and a leading cause of increased mortality and morbidity, in cancer patients (6, 17). Cardiotoxicity can occur in the early or late stages of the course of the disease and may vary from subclinical myocardial dysfunction to irreversible heart failure or death (18). Documented reports are limited to the mechanism of the appearance of cardiac dysfunction during chemotherapy and the susceptibility of patients to develop cardiotoxicity (1, 19). However, a proposed clinical study demonstrated that among all cancer patients, the overall occurrence of DOX-induced cardiotoxicity was ~9%, and most cases occurred immediately during the first year after the completion of chemotherapy and have even been noticed after a follow-up of 4 years (20). Complications emerging from chemotherapy-induced cardiotoxicity are potentially life-threatening, further limiting the clinical use of various chemotherapeutic agents (particularly anthracyclines) (8), thus strongly supporting the need for improved cardioprotective agents.

## Mechanisms Of Dox-Induced Cardiotoxicity

One widely accepted mechanism for DOX-induced cardiotoxicity is the generation of reactive oxygen species (ROS) after DOX treatment in cardiac mitochondria; this occurrence marks as the primary initiating event in the cascade of intracellular modifications (21). In mitochondria, DOX is reduced by NADH dehydrogenase and undergoes redox cycling, generating ROS (22). Elevated levels of ROS result in cellular damage, also known as oxidative stress, which is initiated when the delicate balance between the ROS-generating system and antioxidant measures is disrupted (8). Cardiomyocytes are highly susceptible to oxidative stress, as treatment with DOX reduced the levels of antioxidant enzymes such as glutathione peroxidase, catalase, and superoxide dismutase (23). Cancer patients receiving DOX treatment also undergo immediate systemic oxidative stress, which is due to a decrease in glutathione and total antioxidant capacity of plasma (24).

Production of ROS also affects the DNA, RNA, proteins, and lipids and can also act as secondary signaling molecules in various pathways that are involved in homeostasis, including cell proliferation and cell death (25, 26). Thus, maintenance of a proper level of ROS in the intracellular and extracellular environment is of vital importance. Hence, it could be inferred that oxidative stress could be a leading cause of cellular hypertrophy in the heart (27), due to gene expression alterations (28), cell death activation (29), extracellular matrix transformation (30), ventricular remodeling (29), and calcium transient perturbation (31), all of which could result in the pathophysiological changes that lead to cardiomyopathy and heart failure.

On the other hand, DOX can also disrupt cellular and mitochondrial metabolism, a phenomenon not fully explored. For example, DOX can reduce mitochondrial NADH accumulation and impair oxidative phosphorylation in heart tissues, events associated with reduced glucose uptake (32). Doxorubicin can also induce the opening of mitochondrial permeability transition pore, resulting in the loss of mitochondrial membrane potential, thus explaining DOX-mediated apoptosis in cardiomyocytes. Moreover, DOX can reduce both the protein level and AMPK phosphorylation, thus contributing to stress and metabolic dysfunction (33, 34). More recently, one study found that the noncanonical function of the tumor suppressor p53 is involved in DOX-mediated cardiotoxicity (35). Doxorubicin treatment of *TP53*-depleted mice resulted in left ventricular systolic dysfunction, in association with decreased oxidative metabolism, and reduced mitochondrial volume and DNA transcription. Taken together, induction of oxidative stress and disruption of metabolism in mitochondria are crucial to the development of cardiotoxicity by DOX.

## Role of Epigenetic Alterations in Dox-Induced Cardiotoxicity

**Figure 1:**
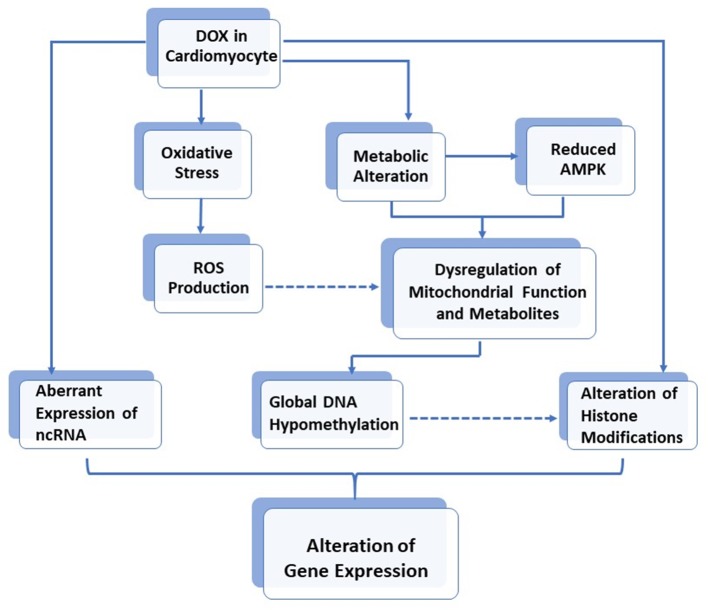
The role of DOX in alteration of gene expressions via epigenetic modifications in cardiomyocytes.

Mitochondrial metabolites constitute a large number of cofactors for several enzymes involved in human biochemical pathways, including epigenetic modifications (36). For example, S-adenosylmethionine (SAM) is the universal substrate for DNA and histone methylation. It is therefore believed that mitochondrial disruption may likely affect cardiomyocyte genomic chromatin (7). Indeed, DNA methylation and histone modifications, as well as non-coding RNA expression, have recently been found to play a role in DOX-induced cardiotoxicity. Furthermore, *in vivo* experiments also demonstrated that rat deficient in methyl donors developed cardiomyopathy with disrupted mitochondrial alignment in the myocardium (37). This effect was due to the reduced activity of PGC-1α, the master regulator for mitochondrial biogenesis (38). Interestingly, such reduced PGC-1α activity was found to be due to increased acetylation and a decreased methylation of PGC-1α, through downregulation of the histone modifiers, SIRT1 deacetylase, and PRMT1 methyltransferase, thus further supporting the interplay between metabolism and epigenetic modifications (37). The role of DOX in the alteration of gene expression via epigenetic modifications is illustrated in Figure 1.

## Epigenetic Modification: DNA Methylation

DNA methylation is often referred to as the “fifth” DNA based, because of its ubiquitousness in occurring at the 5′ position of cytosine in CpG dinucleotide (39). 5-Methylcytosine is established, maintained, and removed by several enzymes, including DNA methyltransferases (DNMTs) and Tet (the ten-eleven translocation hydroxylases) family protein. DNA methylation at the promoter region of a gene is associated with transcriptional repression by recruitment of transcriptional repressors and histone modifiers (such as histone deacetylases and histone methyltransferase), resulting in a repressive chromatin. The interplay between DNA methylation and histone modifications has been reviewed elsewhere (40, 41) and will not be discussed here.

DNA methyltransferases and Tet require SAM or α-ketoglutarate (α-KG) for the formation of 5-methylcytosine or 5-hydroxymethylcytosine (5hMC), in the process of DNA methylation and demethylation, respectively (42). In particular, the metabolic pathway from mitochondria generates SAM and α-KG; mitochondrial dysfunction associated with chronic DOX therapy may affect epigenetic machinery.

Indeed, in one of the studies, mouse cardiomyoblast H9c2 cells were used to analyze the effect of DOX (21). Together with a decrease in glycolytic activity and basal respiration in DOX-treated cells, dysregulation of mitochondrial DNA transcripts was observed. Importantly downregulation of DNMT1 (a maintenance methyltransferase), accompanied by a decrease in global DNA methylation, was also observed. This effect is in agreement with a previous animal study that global DNA hypomethylation, accompanied by a dysregulated expression of mitochondrial gene products encoded from both nuclear and mitochondrial genome, was observed in the hearts of rats treated with DOX (15). It is also interesting to point out that Ferreira et al. (21) found that pre-exposure of DOX can confer resistance to subsequent exposure of DOX in H9c2 cells, probably due to mitochondrial adaptation. As DNA methylation of the mitochondrial genome is maintained by DNMT1, the only DNMT member that can be translocated into the mitochondria (43, 44), downregulation of DNMT1 by oxidative stress may eventually affect the methylation of mitochondrial genome. Taken together, these studies thus suggest that DOX may affect global DNA methylation via dysregulation of mitochondrial function and related metabolites.

Notably, a recent animal study demonstrated that the involvement of mitochondrial genome was not observed, as genes showing significant differential methylation in DOX-treated rats were all encoded from the nucleus. However, a global DNA hypomethylation in the DOX-treated group was still observed (45). This discrepancy may be due to the methods used in these studies. Study from Nordgren et al. (45) utilized a sequencing-based approach Reduced representation bisulfite sequencing (RRBS) that can only interrogate DNA methylation at the CpG rich region, whereas studies from Ferreira et al. used a candidate gene approach to analyze the change of the mitochondrial genome (21). In this regard, further unbiased experiments are required to analyze the role of DOX in the change of the methylome in mitochondria and nucleus.

## Histone Modification

Besides DNA methylation, histone modifications are also involved in DOX-induced cardiotoxicity (Table 1). These modifications can give rise to synergistic or antagonistic interactions with chromatin-associated protein, resulting in dynamic switching between transcriptionally active (accessible euchromatin) and silent (condensed heterochromatin) states (50). For example, histone deacetylase, HDAC6, was found to be upregulated in DOX-treated primary rat cardiomyocytes, *in vitro*, and mice model, *in vivo*, resulting in deacetylation of α-tubulin (48). The upregulation of other HDACs (Table 1) has also been observed in the heart tissue of mice treated with DOX (46). In this regard, Song et al. (48) demonstrated that genetic or pharmacological inhibition of HDAC6 in mice showed a cardioprotective effect against DOX by restoring autophagic flux.

**Table 1:** Changes of histone modifications and modifiers in DOX treated cardiomyocytes.

**Modifiers or Modifications**	**Changes**	**References**
**HISTONE DEACETYLASES**		
HDAC2^1^^,^ ^2^	Downregulated	(46, 47)
HDAC4^1^	Upregulated	(46)
HDAC5^1^	Upregulated	(46)
HDAC6^1^^,^ ^3^	Upregulated	(46, 48)
HDAC7^1^	Upregulated	(46)
HDAC10^1^	Upregulated	(46)
HDAC11^1^	Upregulated	(46)
SIRT1^2^	Contradictory	(47)
**HISTONE LYSINE DEMETHYLASES**		
KDM3A^2^	Upregulated	(47)
LSD1^2^	Downregulated^5^	(47)
**HISTONE LYSINE METHYLTRANSFERASE**		
SET7^2^	Upregulated^6^	(47)
SMYD1^2^	Upregulated^6^	(47)
**HISTONE MODIFICATIONS**		
H3Ac^2^	Downregulated	(47)
Histone^4^	Loss^7^	(49)
H3K4me3^4^	Downregulated^8^	(49)

**Experimental model:**

1 Mice (C57BL/6);

2 H9c2 rat cardiomyocyte;

3 HDAC^−/−^ mice and primary rat cardiomyocyte;

4 mice (unspecified);

5 long term (48 h treatment);

6 high dose and long term (48 h treatment);

7 histone eviction;

8 *downregulation of H34me3 and a shift of peak toward the transcription start site*.

Furthermore, a recent study using H9c2 cardiac myoblast cells also demonstrated that expression of several histone modifiers was dysregulated in association with downregulation of global acetylation of histone H3 (Table 1). In this study, Hanf et al. (47) demonstrated that expression levels of histone deacetylases (SIRT1 and HDAC2) were affected upon DOX treatment. In particular, different isoforms of SIRT1 displayed a contradictory expression level. However, pterostilbene, a natural analog of resveratrol and antioxidant, has been found to alleviate DOX-induced cardiotoxicity both *in vitro* and *in vivo* (51). This effect is due to enhanced deacetylation activity of SIRT1, suggesting its cardioprotective effect against DOX. In the case of HDAC2, treatment with low-dose DOX resulted in decreased expression of HDAC2, but no significant changes in high-dose treatment, as compared to control. Consistently, HDAC2 downregulation was observed in the heart tissue of mice treated with DOX (46). As most of the HDACs were found to be upregulated in DOX-treated cardiomyocytes, it is reasonable to observe the cardioprotective effect of HDAC inhibitors on DOX (52). Intriguingly, studies found that trichostatin A, a pan-HDAC inhibitor, can enhance DOX-mediated hypertrophy and apoptosis in H9c2 rat cardiomyoblasts (53, 54). In one of the studies, Ma et al. (54) found that DOX-induced cardiotoxicity is mediated through Rac1, a GTP-binding protein, and subunit of NADPH oxidase, resulting in the suppression of HDAC activity and upregulation of p53. Importantly, this process is ROS-independent. In this regard, treatment of HDAC inhibitor further enhances the effect of DOX-mediated cardiotoxicity. The involvement of specific HDAC isoforms in this process, however, remains to be determined.

Moreover, the histone lysine demethylase, KDM3A, was significantly upregulated upon DOX treatment of H9C2 cells; however, long-term DOX treatment also significantly decreased the lysine-specific histone demethylase 1 (i.e., LSD1). In parallel, significant upregulation of the histone lysine methyltransferases, SET7 and SMYD1, was only observed in long-term and high-dose DOX treatment. Notably, a heart-specific transcriptional alteration was only observed in mice treated with DOX, but not etoposide, a nonanthracycline (49). This event was due to the inhibition of topoisomerase 2β (55), as “eviction” of specific histones from chromatin, resulting in a shift of histone modification (H3K4me3), and chromatin structure, around the promoter region of a gene.

## Noncoding RNA Expression

Another recognized epigenetic modification is the regulation of ncRNAs, including long noncoding RNAs (lncRNAs) and microRNAs (miRNAs). Noncoding RNAs are involved in numerous human biological processes, as well as human diseases (56). Up to 30% of gene expression in humans is regulated by ~1,000 known miRNAs, ranging from 18 to 25 nucleotides. MicroRNAs may originate from either independent genes or introns of protein coding genes and are transcribed by RNA polymerase II. Subsequently, these “primary miRNAs” are processed into mature miRNAs and then assembled into argonaute family proteins containing ribonucleoprotein complexes called miRNA-induced silencing complexes. These complexes then bind to their mRNA target sequences in 3′ UTR (untranslated region) of mRNA transcripts, resulting in either translational blockage or mRNA degradation.

Aberrant expression of several miRNAs has been shown involved in DOX-mediated cardiotoxicity (Table 2). For example, upregulation of miR-15 was observed in DOX-induced apoptotic H9c2 cardiomyocytes (57). This effect was probably due to suppression of *Bmpr1a*, a target of miR-15 and BMP receptor, previously found to be involved in cardiac contractility (68). Activation of BMP signaling by Bmpr1a agonist is therefore able to rescue DOX-mediated cardiotoxicity in H9c2 cells (57). Similarly, upregulation of miR-23a (58), miR-34a (61, 62), miR-140 (63), miR-146a (64), and miR-532 (66) were observed either *in vitro* or *in vivo* models of DOX-induced cardiotoxicity. Interestingly, upregulation of miR-34a, a well-known tumor suppressive miRNA, could epigenetically suppress *SIRT1* (61, 62), thus partially explaining the downregulation of this HDAC, by DOX, in the aforementioned study (47).

**Table 2:** Expression changes of ncRNA in cardiomyocytes treated with DOX.

**ncRNA**	**Changes**	**Targets**	**Experimental model**	**References**
miR-15b	Upregulated	Bmpr1a, Gata4, Nkx2-5	H9c2 rat cardiomyocyte	(57)
miR-23a	Upregulated	PGC-1α	Rat (Sprague–Dawley); Primary rat cardiomyocyte	(58)
miR-29b	Downregulated	Bax	Rat (Wistar); Primary rat cardiomyocyte	(59)
miR-30	Downregulated	β_1_AR, β_2_AR, Giα-2, BNIP3L	Rat (Sprague–Dawley); primary rat cardiomyocyte; H9c2 rat cardiomyocyte	(60)
miR-34a	Upregulated	Bcl-2, SIRT1	Rat (Sprague–Dawley); H9c2 rat cardiomyocyte	(61, 62)
miR-140	Upregulated	Nrf2, SIRT2	Rat (Sprague–Dawley); mice (C57BL/6); H9c2 rat cardiomyocyte	(63)
miR-146a	Upregulated	ErBb4	Mice (C57BL/6); primary rat cardiomyocyte	(64)
miR-212/132	Overexpression^1^	Fitm2, Sgk3, Rbfox1	Mice (C57BL/6N); primary rat cardiomyocyte; human iPSC-derived cardiomyocyte	(65)
miR-532	Upregulated	ARC	Primary rat and mice cardiomyocyte	(66)
LINC00339	Upregulated	miR-484	Rat (Sprague–Dawley); primary rat cardiomyocyte; H9c2 rat cardiomyocyte	(67)

1 *Adenovirus-mediated overexpression*.

Therapeutically, adenovirus-mediated overexpression of miR-212/132 cluster has been shown to prevent DOX-induced cardiotoxicity in a mouse model (65). This effect may be partially due to direct targeting of *Fitm2*, a transmembrane protein involved in fat storage, by miR-232/132. Moreover, downregulation of miR-29b (59) and miR-30 (60) was also observed in DOX-treated cardiomyocytes in an animal model, leading to de-repression of BAX, a proapoptotic protein, and β-adrenoceptor (β_1_- and β_2_AR), involved in myocyte contraction, respectively.

On the other hand, lncRNAs, which are more than 200 nucleotides long, regulate gene expression by diverse mechanisms (69). For example, lncRNAs can serve as a scaffold to recruit activators or repressors to regulate gene expression. The molecular function and clinical application of lncRNAs in cardiovascular disease have been recently reviewed (70, 71). Particularly, several studies have provided evidence to demonstrate that lncRNA can directly “sponge” or bind to miRNAs, thus regulating the activity of those miRNAs through a competing endogenous RNA (ceRNA) mechanism (71–74). For example, DOX can upregulate the lncRNA, LINC00339 (Table 2), resulting in the suppressing of miR-484 by ceRNA mechanism, in cardiomyocytes *in vitro* and in an animal model (67).

## Conclusion

Although DOX is still the mainstay anthracyclines (9) for the treatment of several human cancers, a major concern is the side effect of cardiotoxicity. In this review, we have summarized recent findings that epigenetic modifications were observed in cardiomyocytes treated with DOX, both *in vitro* and *in vivo*. Although the causal relationship between cardiotoxicity and epigenetic modifications has not been fully explored, epigenetic modifications may contribute to either a cardiotoxic or cardioprotective process. Whether this process is contributed by DOX-mediated ROS or specific signaling pathways may require further investigation (54). Therapeutically, combinations of chemotherapeutic agents with epigenetic therapies, such as small molecule inhibitor of epigenetic writer/reader/eraser or miRNAs manipulations, may confer protection of patients from cardiotoxicity. However, whether such a potential cardioprotective agent will affect the efficacy of DOX or create other side effects requires further clinical investigation (i.e., the colloquial “double-edged sword”). For example, dexrazoxane, the only Food and Drug Administration–approved cardioprotective agent, has been shown to prevent DOX-mediated cardiotoxicity (75). However, the beneficial effect of dexrazoxane is still debated because of the risk for the development of acute myeloid leukemia and myelodysplastic syndrome in children (76, 77). In conclusion, epigenetic modifications may play a role in DOX-mediated apoptosis and atrophy in cardiomyocytes. Delineation of specific epigenetic therapies as detrimental vs. beneficial cardioprotective merits further investigation.

## Author Contributions

W-HH and MC performed literature search. HK and MC wrote the manuscript.

## Conflict of Interest

The authors declare that the research was conducted in the absence of any commercial or financial relationships that could be construed as a potential conflict of interest.
